# Doxorubicin impairs cognitive function by upregulating AMPAR and NMDAR subunit expression and increasing neuroinflammation, oxidative stress, and apoptosis in the brain

**DOI:** 10.3389/fphar.2023.1251917

**Published:** 2023-11-30

**Authors:** Ahmad H. Alhowail, Maha A. Aldubayan

**Affiliations:** Department of Pharmacology and Toxicology, College of Pharmacy, Qassim University, Buraidah, Saudi Arabia

**Keywords:** AMPAR, Bax, COX-2, cognitive impairment, doxorubicin, NMDAR, NF-κB, TNF-α

## Abstract

**Introduction:** The anticancer drug doxorubicin (DOX) is used for various malignancies. However, it also causes cognitive impairment in cancer survivors. In order to determine the mechanisms underlying the acute effects of DOX, we assessed the mRNA and protein expression of glutamate receptors and proteins involved in cognitive function and apoptosis.

**Methods:** Fear-conditioning memory tests were performed in rats after a single intraperitoneal injection of DOX (25 mg/kg) to evaluate short-term memory function. Rat brain samples were collected, and GluA1 mRNA and protein expression; *NR2A* and *NR2B* mRNA expression; and COX-2, NF-kB, TNF-α, and MDA, Bax, and caspase-3 levels were assessed via reverse transcription polymerase chain reaction and enzyme-linked immunosorbent assays.

**Results:** We observed a decreased number of entries in Y-maze, decreased exploration time to the novel object in the novel object recognition (NOR), and decreased freezing time in the fear-conditioning memory tests in DOX-treated rats relative to those in control rats, demonstrating cognitive impairment. *GluA1*, *NR2B*, and *NR2A* expression and MDA, NF-κB, Bax, COX-2, TNF-α, and caspase-3 levels in the brain were significantly elevated in DOX-treated rats.

**Conclusion:** DOX induced cognitive impairment in the rats via neuronal toxicity by upregulating AMPAR and NMDAR expression and increasing neuroinflammation, oxidative stress, and apoptosis in the brain.

## 1 Introduction

The anthracycline doxorubicin (DOX) is widely applied in the therapy of several cancers, including breast and prostate cancers (Thorn et al., 2011). DOX primarily reduces DNA synthesis by inhibiting topoisomerase II, thereby enhancing reactive oxygen species (ROS) production and disrupting mitochondrial function (Alharbi et al., 2020). Although DOX is an effective anticancer agent, it is associated with several side effects, including cardiotoxicity, nephrotoxicity, and hepatotoxicity (Zhao and Zhang, 2017; Song et al., 2019; Afsar et al., 2020; Alharbi et al., 2020). Recent studies have linked DOX to chemo brain (Alharbi et al., 2020). However, only few studies have focused on the underlying mechanisms and etiology of chemo brain (Alhowail et al., 2019a; Alhowail et al., 2019b; Alharbi et al., 2020). DOX is ionized and hence does not easily cross the intact blood–brain barrier (Bredlau et al., 2018). Therefore, it can cause cognitive impairment by directly affecting the central nervous system and through indirect mechanisms, such as excessive ROS production, resulting in increased lipid peroxidation, impaired synaptic plasticity, and mitochondrial dysfunction (Alhowail et al., 2019a). Although DOX impairs hippocampal-dependent tasks and cognitive function, the underlying mechanisms remain unclear.

Glutamate is a prevalent excitatory neurotransmitter and is also neurochemically synthesized in the brain (Zhou and Danbolt, 2014; Lieu et al., 2020). Glutamate receptors are classified as either ionotropic or metabotropic (Crupi et al., 2019). N-methyl-d-aspartate receptors (NMDARs) and -amino-3-hydroxyl-5-methyl-4-isoxazole-propionate receptors (AMPARs) are two examples of ionotropic receptors, which play vital roles in synaptic and cognitive functions (Traynelis et al., 2010; Bhattacharya et al., 2017). Upon neuronal stimulation, the presynaptic terminal releases glutamate into the synaptic cleft, which binds to AMPARs and NMDARs and causes conformational changes (Alhowail et al., 2022). AMPARs elicit an influx of Na^+^, depolarizing the membranes of postsynaptic neurons (Zanetti et al., 2021; Alhowail et al., 2022). At resting potential (−65 mV), NMDARs are closed by Mg^2+^ (Kampa et al., 2004). Postsynaptic membrane depolarization by AMPARs removes this blockade, permitting Ca^2+^ influx, which is essential for downstream memory formation (Luscher and Malenka, 2012). AMPARs comprise four subunits (GluA1–4), which are differentially expressed (Bhattacharya et al., 2017; Polli and Kohlmeier, 2022). In the adult brain, GluA1 is permeable to Ca^2+^; however, GluA2 is not permeable to Ca^2+^ because of the presence of arginine in its receptor, which blocks Ca^2+^ permeability through AMPARs (Wright and Vissel, 2012; Livingstone et al., 2021). Therefore, the presence of more GluA1 in the AMPAR structure allows Ca^2+^ influx through the cell membrane, causing neuronal toxicity (Qu et al., 2020; Livingstone et al., 2021). In addition, NMDARs comprise the NR1, NR2A, NR2B, and NR2C subunits, which are vital to synaptic plasticity and cognitive function (Brothwell et al., 2008; Suzuki et al., 2023). Therefore, alterations in the expression and activity of these receptors directly affect general brain function by reducing or increasing downstream signaling, thereby causing cognitive impairment (Yang et al., 2022).

The transcription factor nuclear factor kappa B (NF-κB) controls several genes related to cellular development, survival, apoptosis, and stress response as well as inflammation development and progression (Park and Hong, 2016). Activation of NF-κB can stimulate the transcription of TNF-α genes, which activate tumor necrosis factor receptor 1 (TNFR1), promoting proinflammatory cytokine production and leading to neuroinflammation, upregulation of cyclooxygenase-2 (COX-2) expression, and apoptosis (Poligone and Baldwin, 2001; Li et al., 2005). Furthermore, the pro-apoptotic Bcl-2-associated X protein (Bax) is a mitochondrial membrane protein that regulates membrane permeability (Singh et al., 2015; Hsieh et al., 2016). At the physiological cellular level, Bax expression and function regulate the mitochondrial membrane (Shamas-Din et al., 2013). However, under oxidative stress, the increase in Bax expression in the outer mitochondrial membrane induces the formation of pores, which cause the release of cytochrome C, activating the apoptosome and caspase-3 cascade, resulting in apoptosis (Gao and Wang, 2009; Naseri et al., 2015). Moreover, increased malondialdehyde (MDA), ROS production, and lipid peroxidation characterize oxidative stress, resulting in damage to cellular function, and stimulate apoptosis (Su et al., 2019). The susceptibility of the brain to oxidative stress and its effects on metabolism and synaptic activities have been reported in Parkinson’s disease and Alzheimer’s disease (Pimentel et al., 2012). In addition, increasing evidence suggests that DOX can induce cognitive decline, reduced long-term potentiation (LTP), and synaptic dysfunction (Alhowail et al., 2019a).

Decreased expression and activity of AMPARs and NMDARs can impair cognitive function (Li and Tsien, 2009; Alhowail, 2021). In contrast, upregulated AMPAR and NMDAR expression increases Ca^2+^ influx, causing neuronal injury, toxicity, and apoptosis, ultimately leading to cognitive dysfunction (Newcomer et al., 2022). Therefore, cellular Ca^2+^ balance is essential for memory formation (Ureshino et al., 2019). Despite the high incidence of chemo brain in breast cancer survivors and widespread use of DOX in breast cancer therapy, little is known about the effects of DOX on cognitive function in female rats. Thus, in this research, it is investigated DOX-induced cognitive impairment in female rats. The goal of this investigation was to determine the acute effects of DOX on cognitive function, particularly its impact on the expression of AMPAR and NMDAR subunits; inflammatory and oxidative stress mediators, such as NF-κB, COX-2, MDA, and TNF-α; and markers of apoptosis, such as caspase-3 and Bax.

## 2 Materials and methods

### 2.1 Chemicals and drugs

DOX was purchased from EBEWE Pharma GmbH Nfg KG (Attersee, Austria).

### 2.2 Animal treatments

Twenty female albino rats, each aged 3 months, were housed individually in small cages. These cages were subjected to a light/dark cycle of 12 h per day, with the lights being switched on precisely at 7:30 a.m. Throughout the duration of the study, the rats were provided with unrestricted access to both water and food. The experimental subjects, namely, the rats, were divided into two distinct groups for the purpose of this study. The first group, referred to as the control group, consisted of ten rats. Similarly, the second group, known as the DOX group, also comprised ten rats. The DOX group was administered intraperitoneal (i.p.) injections of 25 mg/kg DOX, while the control group was subjected to a solitary i. p. injection of saline.

### 2.3 Y-maze

**FIGURE 1:**
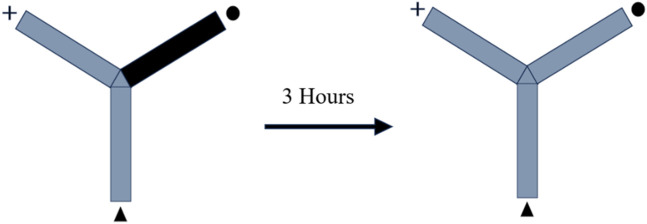
Illustration depicting the schematic representation of the experimental design.

The Y-shaped maze has three wooden arms that measure 50 cm, 10 cm, and 20 cm, respectively, and they are arranged at an angle of 120°. In order to evaluate the rats’ memory, the novel arm of the Y-maze was purposefully made inaccessible. In this particular study, each arm was categorized as either a “starter,” “familiar,” or “novel.” One rat was put into the starting arm, and during each training session, it had free access to the familiar arm for a period of 10 minutes. The test was redone after 3 hours with no restrictions placed on maze exploration and all arms open. For the second time during the experiment, the animal was positioned in the starter arm, and its behavior during the subsequent 3 minutes was monitored to determine whether it preferred the unfamiliar or the familiar conditions. Within the confines of the maze, light was spread out uniformly. It was determined by analyzing videos of the test sessions how long rats remained in the novel arm as well as how many times they entered the arm. It was determined that the animal had entered the arm if all four paws were seen entering the arm (Figure 1) (Alsaud et al., 2023).

### 2.4 Novel object recognition test

**FIGURE 2:**
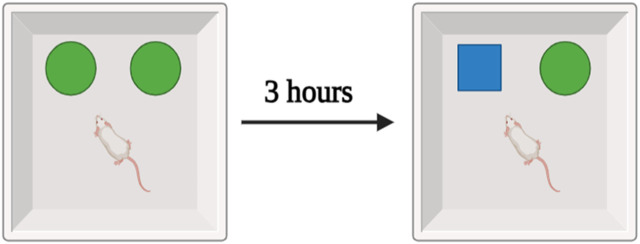
Schematic representation of the experimental design.

In the experiment, a wooden box measuring 40 cm on a side, 40 cm on a side, and 40 cm on a side was used to contain two items: a set of familiar black cans, and an unfamiliar white teacup. The rat spent 10 minutes in the middle of the maze investigating a group of similar black cans. The training period lasted for 3 hours, and then the test session consisted of exposing the rat to a new object (a teacup) for 3 minutes. During the testing session, video cameras recorded the amount of time spent by each animal investigating a novel object. The collected data were then subjected to statistical analysis (Figure 2) (Alsaud et al., 2023).

### 2.5 Fear-conditioning memory tests

**FIGURE 3:**
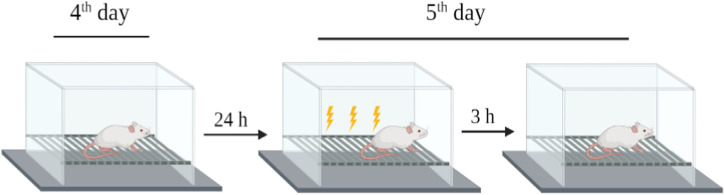
Schematic of the experimental design for fear-conditioning memory test.

The rats were placed in a standard rat operating chamber in a sound isolation cubicle with an electrified grid floor to deliver shocks to their feet (hereafter referred to as “the context”). A conditioned freezing protocol was used to train the rats. On day 4 after treatment, the rats were exposed to the context for 30 min to habituate them to the chamber without foot shock. On day 5 after treatment, the rats were returned to the chamber for 180 s and received multiple electrical foot shocks in different contexts. After 3 h (day 5), the rats were restored to baseline for 180 s without electrical foot shocks. Freezing behavior (no movements except respiration-related movements) was used to evaluate fear memory function by analyzing the changes in freezing times between the treated and control groups (Figure 3) (Alhowail et al., 2022).

### 2.6 Preparation of brain samples

The rodents were humanely euthanized via CO2 inhalation, followed by the decapitation of their heads prior to the extraction of their brains. Following the removal of blood from the brains utilizing phosphate-buffered saline (PBS), the subsequent step involved the extraction of the brain. In order to facilitate the extraction of proteins from neurons, we utilized a Qsonica homogenizer (operating at a frequency of 30 Hz) manufactured by the esteemed company located in Newtown, CT, United States of America. Additionally, we employed a lysis buffer (specifically, N-PER) that was procured from the reputable supplier Thermo Scientific, headquartered in Madison, WI, United States of America. The specimens were subjected to centrifugation at a temperature of 4 °C for a duration of 10 min at a force of 12,000 times the acceleration due to gravity (12,000 × g). Following centrifugation, the resulting liquid portion above the sediment, known as the supernatant, was carefully transferred to newly prepared Eppendorf tubes. Prior to subjecting the samples to enzyme-linked immunosorbent assay (ELISA), the bicinchoninic acid (BCA) assay was conducted in order to ascertain the total protein content (Alhowail et al., 2019a).

### 2.7 Reverse transcription polymerase chain reaction (RT-PCR)

The RNA was isolated from brain samples obtained from animals treated with DOX and control animals utilizing the TRIzol reagent, a product manufactured by Sigma-Aldrich located in St. Louis, MO, United States of America. The residual genomic DNA was subsequently isolated from the total RNA using RNase-free DNase (Ambion, Carlsbad, CA, United States of America). The RNA content and absorbance were calculated utilizing a NanoDrop spectrophotometer manufactured by Thermo Fisher Scientific, located in Loughborough, United Kingdom. The synthesis of complementary DNA (cDNA) was performed using a cDNA synthesis kit (Applied Biosystems, Foster City, CA, United States of America) on a total RNA sample of 500 ng. The cDNA samples were subsequently amplified using Taq DNA polymerase (Qiagen, Shanghai, China). SYBR Green, a fluorescent dye commonly employed in molecular biology research, specifically in the realm of reverse transcription polymerase chain reaction (RT-PCR), was utilized in this study. The RT-PCR procedure was conducted on an iCycler iQ5 system, a thermocycler manufactured by Bio-Rad, a reputable company based in Hercules, California, United States. The synthesis of primers was performed utilizing a proprietary software developed by Integrated DNA Technologies. The RT-PCR experiment was performed utilizing Bio-Rad’s Advanced SYBR Green Supermix with the specified parameters: an initial denaturation step at 95°C for 30 s, followed by 40 amplification cycles consisting of denaturation at 95°C for 5 s, and annealing/extension at 57°C for 30 s. Duplicate samples were meticulously prepared, and a rigorous triad of tests was conducted. After the completion of plate-setting, the acquired data were subjected to automated processing utilizing the AiraMx software for the purpose of comparative quantification. The gene expression levels were standardized relative to the reference gene *GAPDH*, which is commonly used as a housekeeping gene. The quantification of mRNA expression alterations was determined through the estimation of transcript abundance per gene in relation to the reference gene *GAPDH* (Table 1).

**TABLE 1:** Primers utilized in the study.

Gene	Sequence (5′–3′)	Length (bp)
GluA1	Forward: GCC​AGA​TCG​TGA​AGC​TAG​AAA	80
GluA1	Reverse: CTC​CGC​TCT​CCT​TGA​ACT​TAT​T	
NR2A	Forward: GGA​GGA​GGT​TGG​GTC​ATT​TAT	86
NR2A	Reverse: AGT​AGG​CAC​TTG​GGA​CTT​TAC	
NR2B	Forward: GAG​GAA​CCA​GGC​TAC​ATC​AAA	83
NR2B	Reverse: GGT​CAC​CAG​GTA​AAG​GTC​ATA​G	
GAPDH	Forward: ACT​CCC​ATT​CTT​CCA​CCT​TTG	104
GAPDH	Reverse: CCC​TGT​TGC​TGT​AGC​CAT​ATT	

### 2.8 ELISA

Brain samples from the rats in the DOX and control groups that were treated with N-PER and used for BCA were subjected to ELISA for GluA1, Bax, and caspase-3 levels using commercially available kits (MyBioSource Company, San Diego, CA, United States of America) according to the protocols of the manufacturers. The absorbance at 450 nm was read using a BIO-TEK Absorbance Microplate Reader (BioTek, Winooski, VT, United States of America). The data were then subjected to statistical analysis.

### 2.9 Statistical analysis

Data from DOX-treated and control rats were compared utilizing a two-tailed unpaired Student’s t-test in GraphPad Prism 10.0.0.153 (GraphPad Prism Software, San Diego, California, United States of America). When p was smaller than 0.05, a difference was found using every parametric statistic. Both the mean and the standard error of the mean (SEM) are shown for these results.

## 3 Results

### 3.1 DOX increases the mortality rate of rats

**FIGURE 4:**
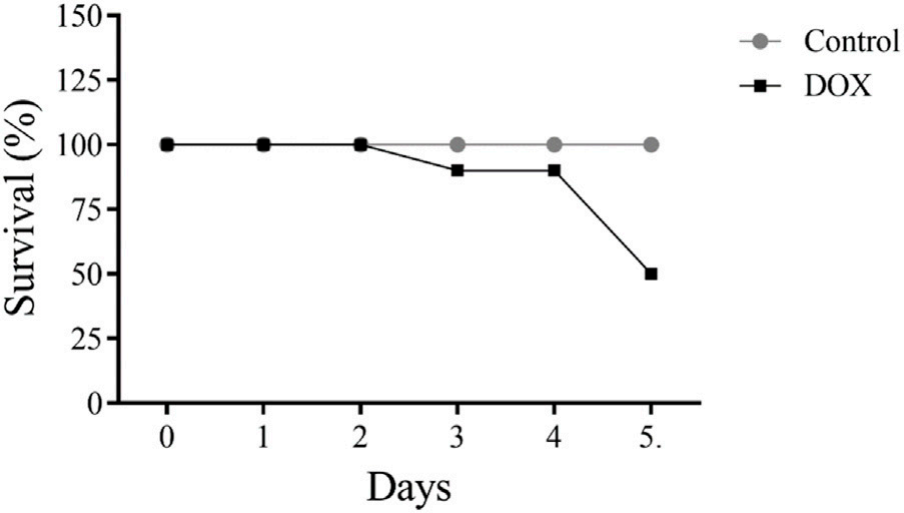
Effects of acute doxorubicin (DOX) treatment on rat survival. Only 50% of rats in the DOX group survived after 5 days of treatment.

5 of 10 (50%) rats administered DOX died after 5 days of therapy (Figure 4).

### 3.2 DOX decreases body weight of rats

**FIGURE 5:**
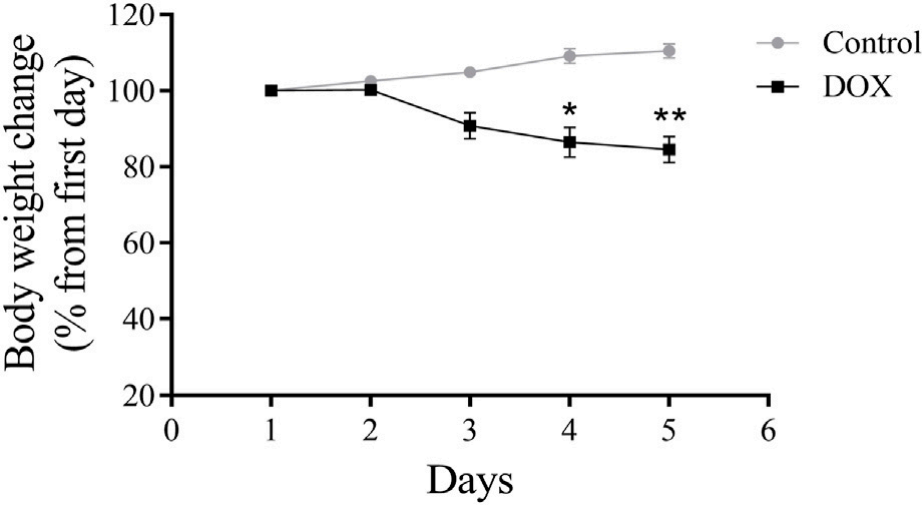
Effects of DOX on rat body weight. The study lasted for 5 days. The DOX-treated rats had significantly reduced body weights. *p < 0.05, **p < 0.01.

The body weight of DOX-treated rats was substantially lower than that of control rats (Figure 5).

### 3.3 DOX impair cognitive function in the Y-maze test

**FIGURE 6:**
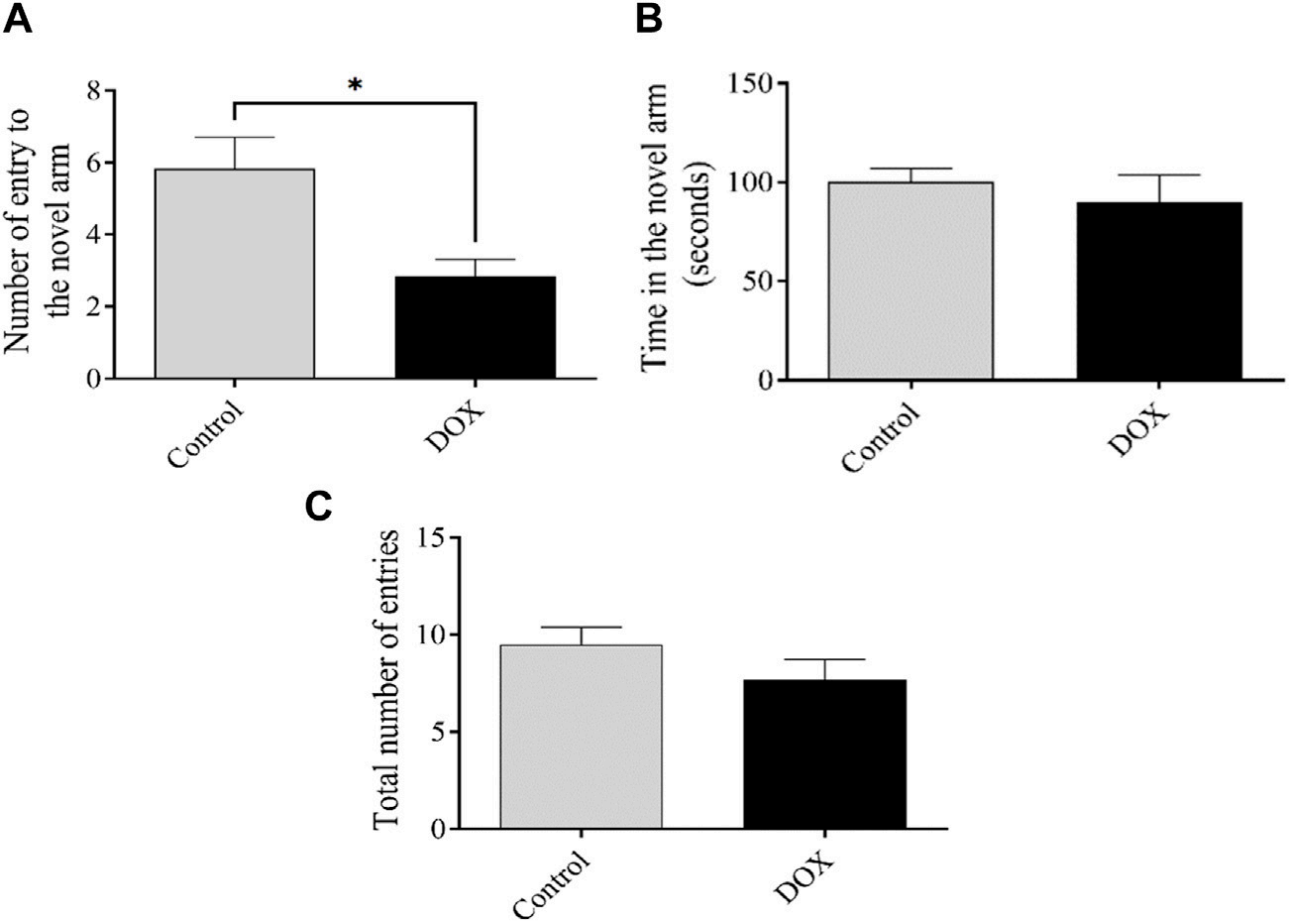
The impact of DOX on rat behavior in the Y-maze test shall be elucidated. **(A** and **B)** The impact of DOX administration on the quantity of entries and duration of stay in the novel arm. **(C)** The cumulative count of participants across all treatment groups. The data presented in this study is represented by bars, which indicate the mean value plus or minus the standard error of the mean (SEM). (*p < 0.05).

The number of entries and time spent in the novel arm reflect the ability of an animal to discover a new unexposed arm. The quantity of rat entries within the DOX group exhibited a statistically notable drop (*p* < 0.05) when related to the control group’s rat entries. (Figure 6).

### 3.4 Effect of DOX on rat behavior in the novel object recognition test

**FIGURE 7:**
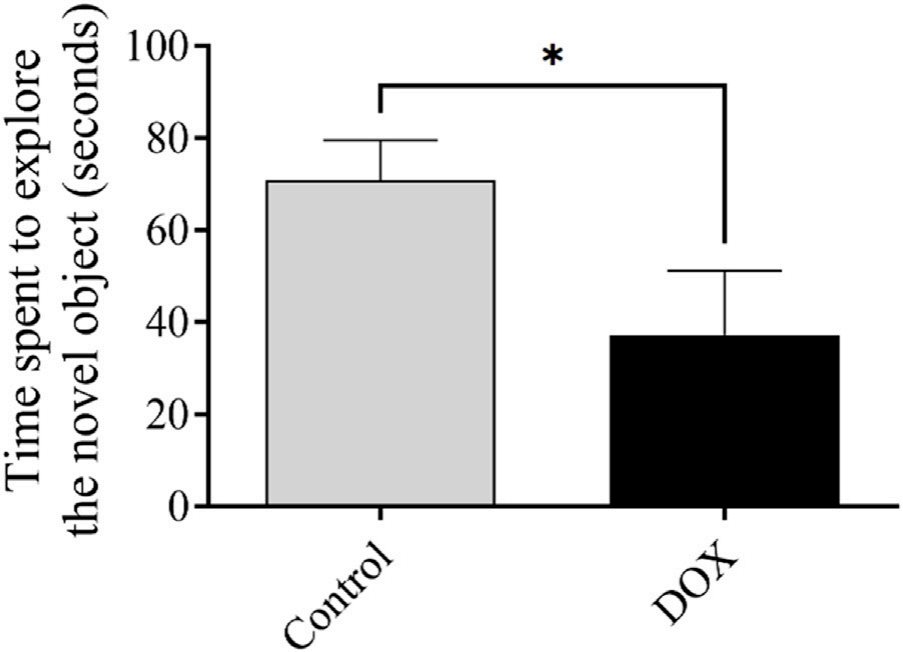
The impact of DOX on rat behavior within the context of the novel object recognition test. The impact of DOX administration on the duration dedicated to the investigation of the novel object. The data is represented by bars, which indicate the mean value plus or minus the standard error of the mean (SEM) (*p < 0.05).

The exploration time reflects the ability of an animal to recall a previously exposed object. The exploration time of rats in the DOX group was significantly lower (*p* < 0.05) that of the control rats (Figure 7).

### 3.5 Effect of DOX on rat behavior in the fear-conditioning memory test

**FIGURE 8:**
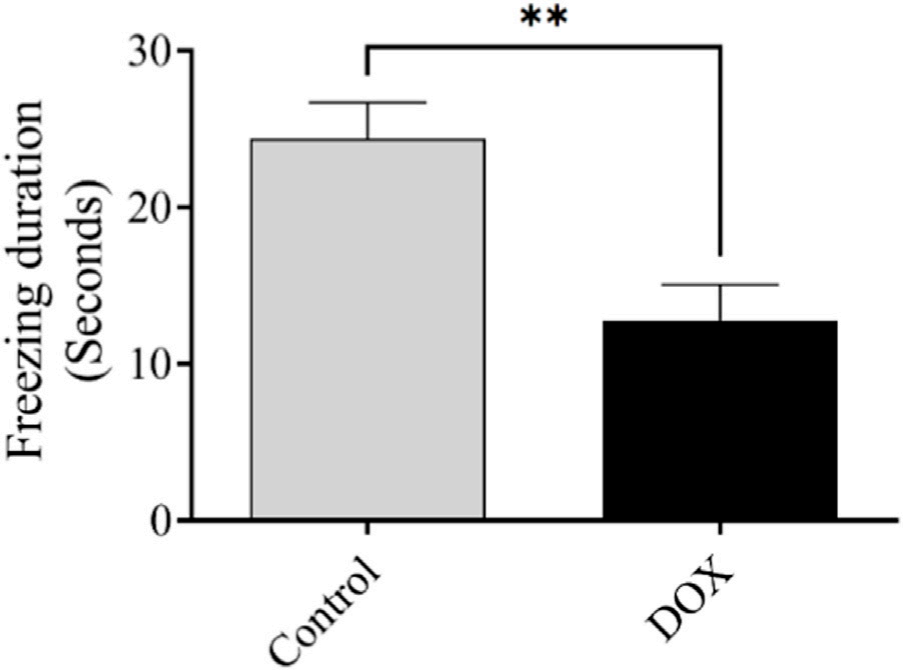
Effect of DOX on rat behavior in the fear-conditioning memory test. Effects of DOX treatment on freezing time. Bars indicate the mean ± SEM (**p < 0.01).

The freezing time reflects the ability of an animal to recall a previously exposed context. The freezing time of rats in the DOX group was notably lower (*p* < 0.01) than that of the control rats (Figure 8).

### 3.6 DOX therapy elevates mRNA and protein expression of GluA1 of AMPARs

The assessment of mRNA expression pertaining to GluA1-containing AMPARs was conducted subsequent to a 5-day administration of DOX treatment. As depicted in Figure 6, the rats subjected to DOX treatment demonstrated a notable elevation in the levels of GluA1 mRNA expression in comparison to the levels observed in the control rats.

### 3.7 DOX upregulates the mRNA expression of NMDAR subunits NR2A and NR2B

**FIGURE 9:**
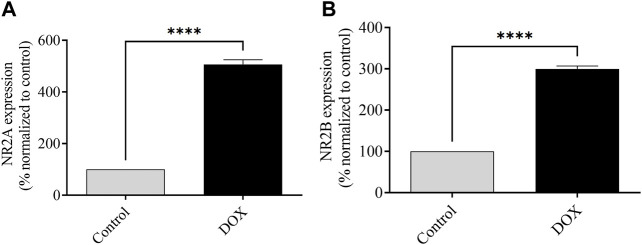
The impact of DOX on the mRNA expression of NR2A and NR2B, in comparison to the levels observed in control rats, is being evaluated. The expression of NR2A mRNA was observed to be significantly elevated in the group treated with DOX in comparison to the control group. The rats that received DOX treatment displayed an increase in the expression of NR2B mRNA, similar to the levels observed in the control rats. The utilization of bars in this context indicates the representation of the mean value along with the standard error of the mean (SEM) (****p < 0.0001).

The levels of NR2A and NR2B mRNA expression were assessed 5 days after DOX administration. The DOX-treated rats exhibited a significant elevation of *NR2A* and *NR2B* mRNA expression compared to those in control rats, which indicated the potential toxic effects of DOX (Figure 9).

### 3.8 DOX upregulates NF-κB, MDA, COX-2, and TNF-α expression

**FIGURE 10:**
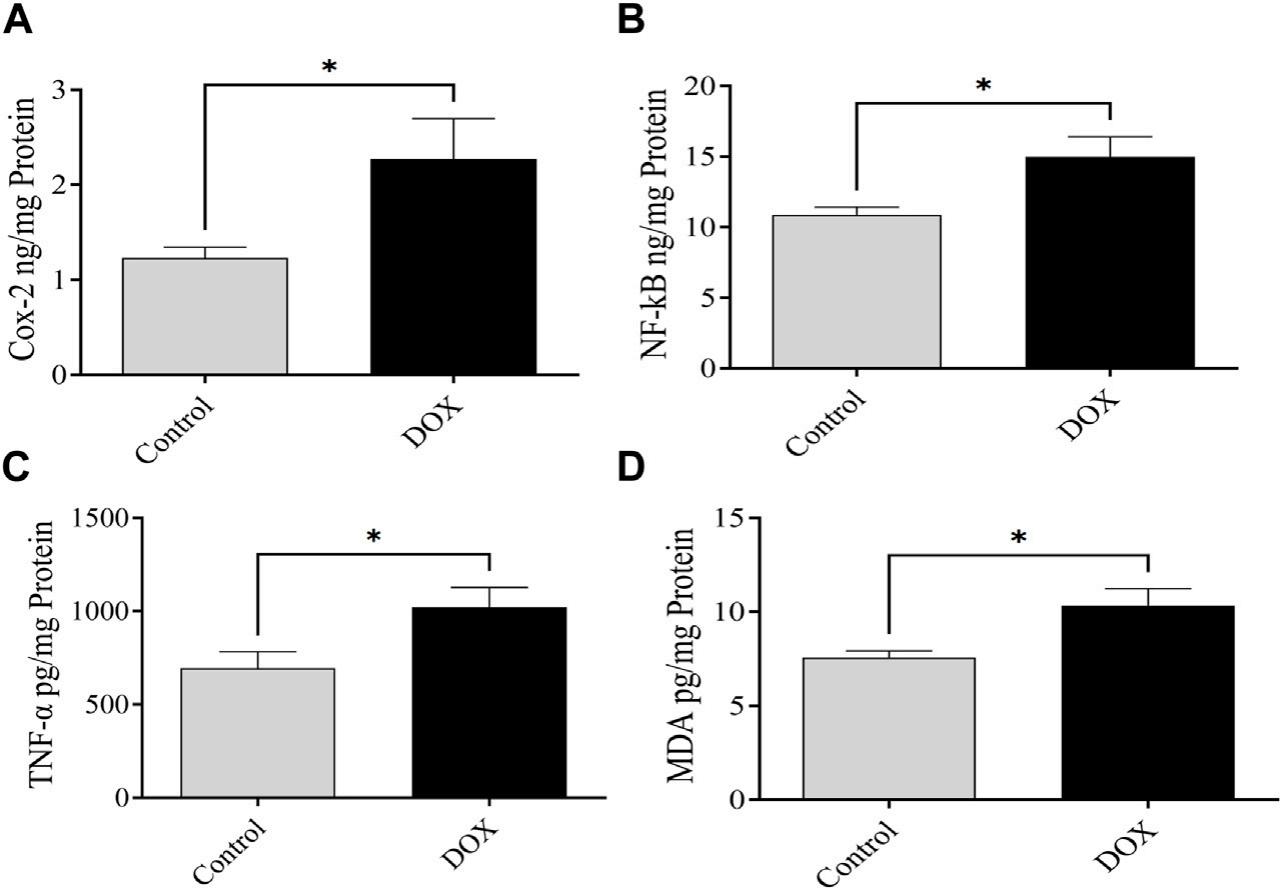
Effects of DOX on the expression levels of NF-κB, COX-2, MDA, and TNF-α in the brain. **(A–D)** DOX-treated rats showed significantly higher COX-2 **(A)**, NF-κB **(B)**, TNF-α **(C)**, and MDA **(D)** expression than the controls. Bars symbolize the mean ± SEM (*p < 0.05).

The expression levels of NF-κB, MDA, COX-2, and TNF-α were assessed 5 days following DOX administration. The brain of DOX-treated rats demonstrated significantly increased levels of NF-κB, COX-2, MDA, and TNF-α compared with that of the controls (Figure 10).

### 3.9 DOX upregulates Bax and caspase-3 expression

**FIGURE 11:**
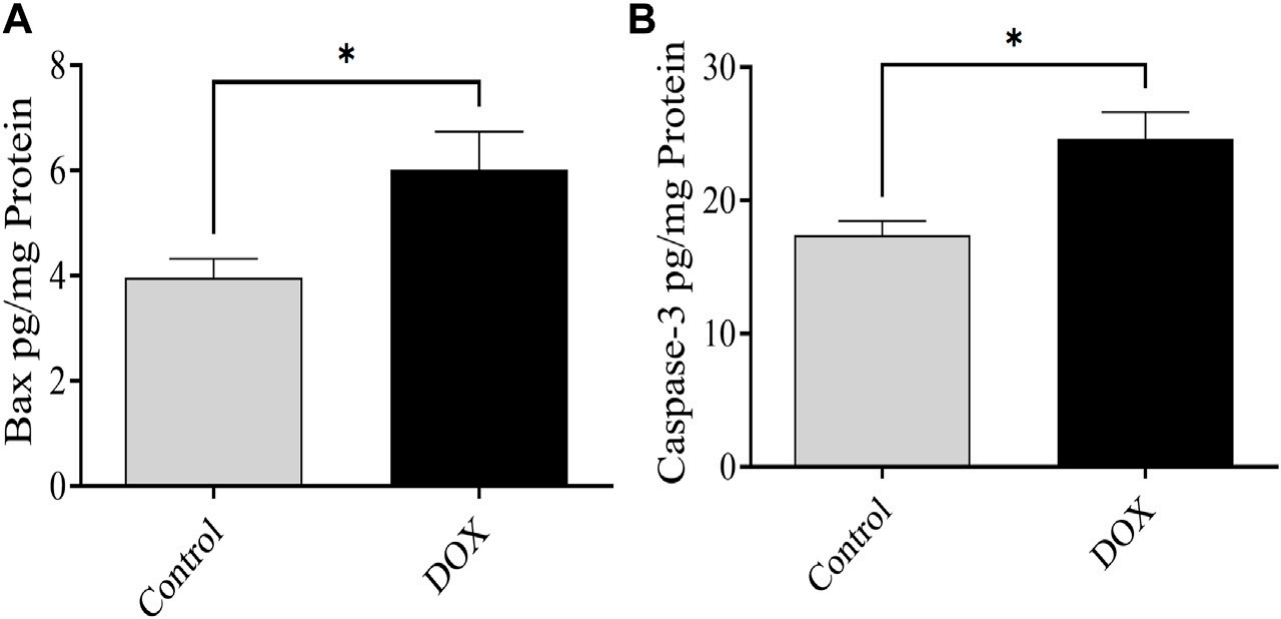
Effects of DOX on caspase-3 and Bax levels in the brain. **(A and B)** The DOX-treated group showed significantly higher Bax **(A)** and caspase-3 **(B)** expression levels than the control group. Bars indicate mean ± SEM (*p < 0.05).

The expression of Bax and caspase-3 was evaluated 5 days after DOX therapy. Rats in the DOX group exhibited a prominent elevation in the levels of caspase-3 and Bax in the brain compared with the controls (Figure 11).

## 4 Discussion

In this study, we observed the impact of DOX on glutamatergic system-induced neuronal toxicity and cognitive impairment in a rat model. The results revealed that DOX caused neurotoxicity via overactivation of the glutamatergic system, thereby inducing apoptosis. DOX administration also significantly reduced body weight, accompanied by a decrease in spatial memory, as evidenced by a reduction in the number of entries in the Y-maze. Additionally, DOX administration resulted in a decrease in exploration time in the NOR test, a decline in freezing time in the fear-conditioning memory test, significantly elevated levels of the mRNA/protein expression of the glutamate subunit of AMPARs containing GluA1, and elevated mRNA expression levels of the glutamate subunit of NMDARs, which together resulted in neurotoxicity and memory impairment. Previous studies have shown that chronic DOX treatment causes cognitive impairment. One proposed mechanism involves reduced hippocampal neurogenesis (Kitamura et al., 2015; Alharbi et al., 2020; Usmani et al., 2023). The Y-maze and novel object recognition tests, in addition to elevated plus maze tests, in our previous research, have also shown that chronic DOX treatment impairs memory function (Alharbi et al., 2020; Alsaud et al., 2023). In this study, we investigated acute DOX treatment by evaluating spatial memory impairment using the Y-maze and NOR tests and other methods of memory impairment by assessing fear conditioning memory, which functions through a distinct pathway (amygdala-dependent learning memory), and the involvement of AMPARs and NMDARs in cognitive impairment as well as neuroinflammation, oxidative stress, and apoptosis.

**FIGURE 12:**
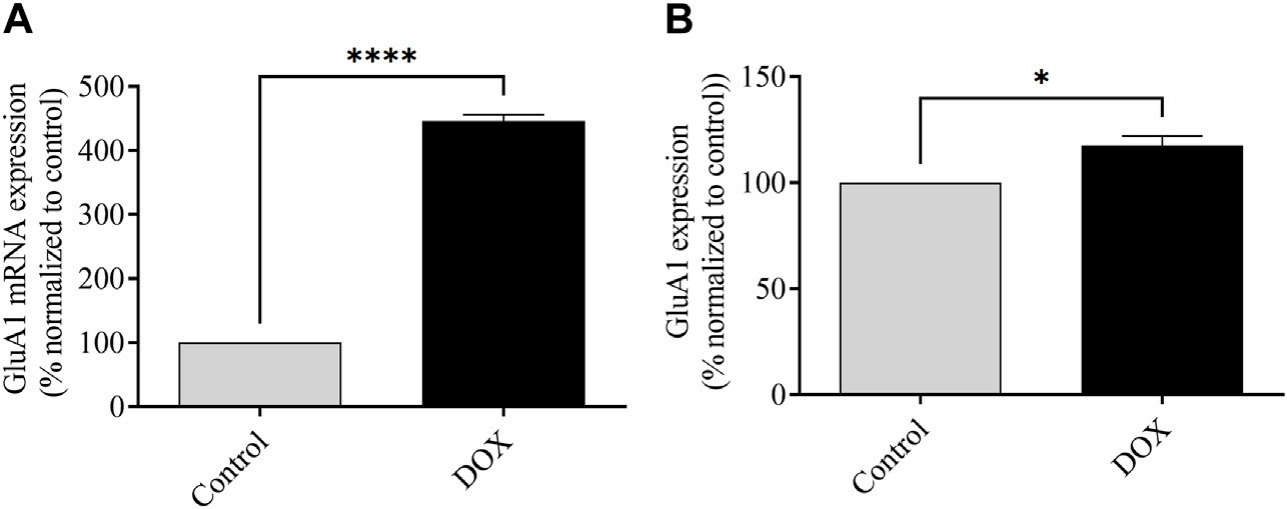
Effects of DOX on GluA1-containing AMPAR expression. **(A)** Effects of DOX on mRNA expression of the GluA1 subunit of AMPARs relative to that in control rats. **(B)** Effects of DOX on AMPAR GluA1 subunit protein expression relative to that in control rats. Bars indicate mean ± SEM (*p < 0.05, ****p < 0.0001).

Alterations in AMPA and NMDA receptors can cause changes in neuronal function, which in turn alters the function of the central nervous system and can lead to cognitive impairment (Li and Tsien, 2009; Alhowail, 2021). These results support the hypothesis that DOX induces cognitive impairment in patients administered DOX. The GluA1 subunit protein and mRNA expression was upregulated in the brains of DOX-treated rats, which caused overactivation of neurons, increased Ca^2+^ influx, and increased Ca^2+^ concentration, leading to neuronal toxicity. Furthermore, our latest research involving the hippocampus of nude mice has uncovered the detrimental effects of chronic (Figure 12) DOX treatment on cognitive function. It was observed that this treatment led to a decrease in the presence of GluA1 subunit-containing AMPA receptors, which are crucial for proper brain function (Alhowail et al., 2021). Nevertheless, the evaluation of the current study, which utilized an acute dose of DOX, demonstrated a noteworthy rise in the presence of the GluA1 subunit within the AMPA receptor. Thus, it is speculated that the variation in results may be attributed to the utilization of the entire brain for protein expression assessment. Furthermore, the initiation of DOX treatment leads to an increase in GluA1, which in turn triggers apoptosis. Nevertheless, extended periods of treatment may lead to neuronal degeneration.

In addition, NMDARs play a vital role to synaptic plasticity and cognitive function (Brothwell et al., 2008; Suzuki et al., 2023). In cases of prenatal nicotine or alcohol exposure, NMDAR expression is reduced, resulting in memory deficits and impaired LTP (Alhowail et al., 2021; Alhowail, 2022; Polli and Kohlmeier, 2022). Similarly, blocking NMDAR expression reduces LTP induction and results in memory impairment (Yang et al., 2018). In contrast, NMDAR overactivation can increase Ca^2+^ entry into neurons, leading to neuronal toxicity and initiation of apoptosis (Dong et al., 2009; Ndountse and Chan, 2009). These results agree with a recent finding that post-treatment with DOX can elevate glutamate levels owing to decreased clearance in the frontal cortex of rodent models (Thomas et al., 2017), which increases glutamate receptor expression.

DOX administration leads to enhanced expression of proinflammatory mediators, including TNF-α, in the brain (Keeney et al., 2018). NF-κB activates the TNF-α gene to enhance the transcription and translation of proteins (Coelho-Santos et al., 2015). The proinflammatory cytokine TNF-α may cross the blood–brain barrier and result in central inflammation in the brain (Huang et al., 2020). The results of the current study revealed that DOX causes neurotoxicity by enhancing the production of proinflammatory mediators TNF-α and COX-2 in the brain, leading to neuroinflammation. These results were evident from the significantly elevated levels of TNF-α, NF-κB, and COX-2 in the brain samples from the DOX-treated rats compared to those in the controls. Furthermore, the DOX-induced elevated neuroinflammation was linked to oxidative stress due to increased levels of MDA in the brain, causing cognitive impairment.

Furthermore, it is imperative to note that the adequate expression and optimal functioning of Bax are indispensable for maintaining the regular cellular processes and promoting proper cellular development (Hardwick and Soane, 2013). Bax, an indispensable participant in the process of apoptosis, assumes a crucial function in the activation of caspase signaling through the release of cytochrome C from the mitochondria (Wang and Youle, 2009). The upregulation of Bax expression elicits the release of cytochrome C, thereby instigating the process of apoptosis through the activation of caspase-3 (An et al., 2004; Mizuta et al., 2007). Moreover, the pathogenesis of various disorders, including Alzheimer’s and Parkinson’s disease, can lead to the upregulation of Bax and caspase-3 expression, ultimately resulting in neurodegeneration (Long et al., 2021; Wolfrum et al., 2022). In a similar vein, chemotherapeutic agents, such as DOX, have been observed to enhance the expression of Bax and caspase-3, leading to the induction of apoptosis (Sharifi et al., 2015; Singh et al., 2019). The results of the present study revealed notable differences in the expression levels of caspase-3 and Bax between the group treated with DOX and the control group. Specifically, it was observed that these levels exhibited an increase subsequent to DOX therapy. Hence, the administration of acute DOX therapy has the potential to enhance the process of apoptosis through the upregulation of Bax and caspase-3 expression, ultimately leading to the initiation of apoptosis. Additional investigations pertaining to Bax and caspase-3, along with their associated signaling proteins situated both upstream and downstream, including extracellular signal-regulated kinase and the generation of reactive oxygen species, are imperative in order to gain comprehensive insights into the modifications induced by DOX in the expression of Bax and caspase-3.

This study is subject to specific strengths and limitations. To the utmost extent of our comprehension, this investigation represents the primary endeavor to combine the impact of DOX on the intricate interplay of oxidative stress, neuroinflammation, and the excessive stimulation of the glutamatergic system. The animal subjects utilized in this investigation were of identical strain and age, and all experimental procedures were carried out concurrently across the study cohorts to mitigate the influence of confounding variables. Additionally, the utilization of cancer-free rats was employed to assess the direct impact of DOX treatment, thereby minimizing any potential confounding effects originating from the presence of cancer. Moreover, it should be noted that one aspect of concern relates to the administration of a solitary dose to the animal subjects, consequently resulting in a partial replication of the dosing regimen observed in human individuals. However, the selection of this specific dosage was made with the intention of investigating its impact on survival rates and the underlying mechanisms of cognitive impairment. An additional limitation concerns the exclusive evaluation of mRNA levels pertaining to NMDARs subunits NR2A and NR2B, without simultaneous examination of protein expression in the brain. However, it is important to note that the aforementioned situation can be ascribed to the inherent limitations imposed by the existing laboratory infrastructure.

**FIGURE 13:**
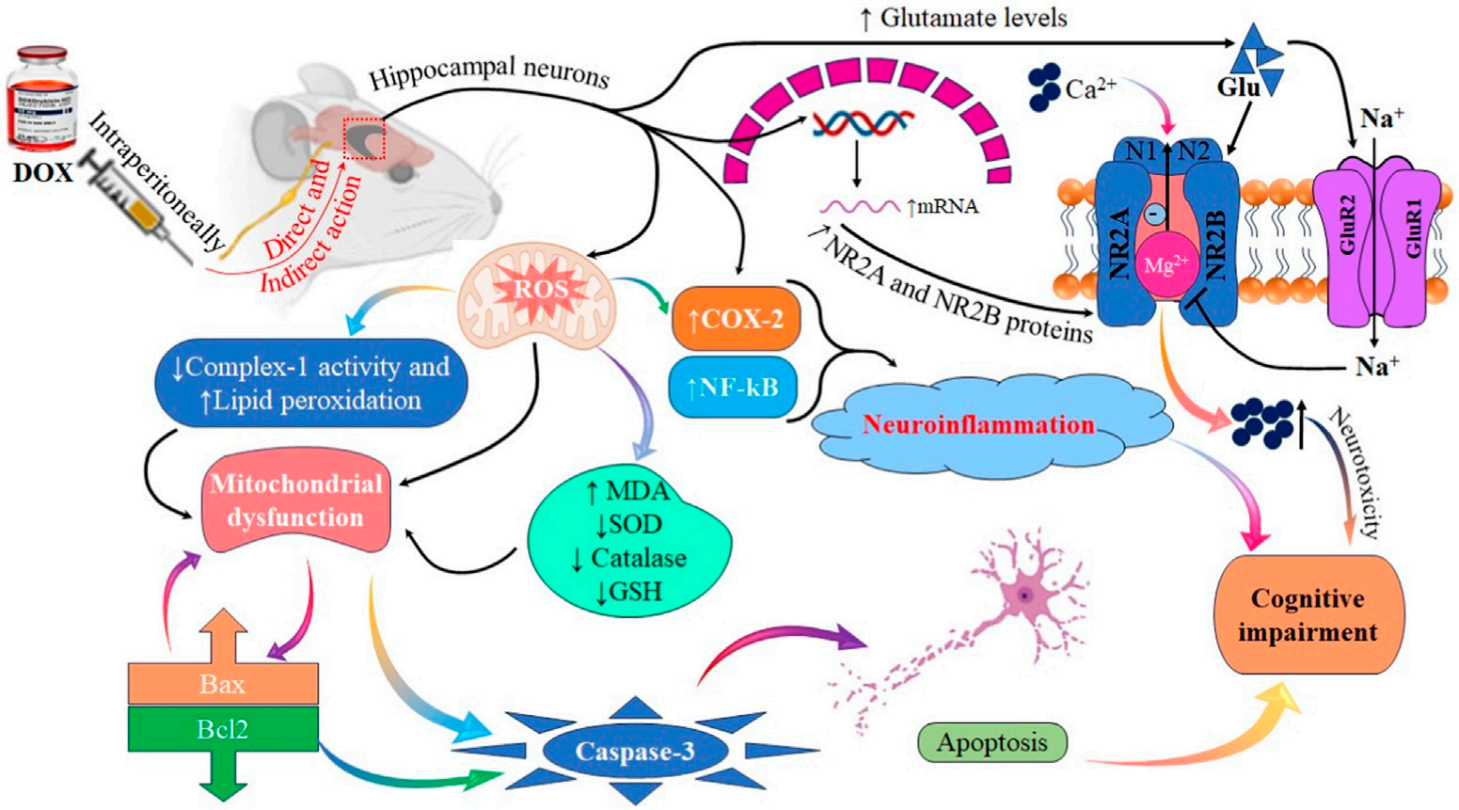
Illustrative figure demonstrating the effects and mechanism of DOX in causing cognitive impairment and neurotoxicity.

In conclusion, our findings supported the hypothesis that DOX induces cognitive impairment by altering glutamate receptor expression, leading to neurotoxicity. Furthermore, the molecular mechanism underlying cognitive impairment was investigated, and the results suggested that DOX increased the expression of the GluA1 subunit of AMPARs and *NR2A* and *NR2B* subunits of NMDARs. This was associated with neuronal toxicity via activation of inflammatory and oxidative stress mediators, such as NF-κB, COX-2, MDA, and TNF-α, and pro-apoptotic protein (Bax, and caspase-3) signaling, which resulted in apoptosis and decreased cognitive performance. Additional research is required to examine the expression and function of AMPAR and NMDAR subunits following acute DOX exposure. A comprehensive understanding of the mechanisms underlying chemotherapy can help elucidate the pharmacological management required to mitigate the cognitive impairment caused by chemotherapy (Figure 13).

## Data Availability

The original contributions presented in the study are included in the article/supplementary material, further inquiries can be directed to the corresponding author.

## References

[B1] Afsar T. Razak S. Almajwal A. Al-Disi D. (2020). Doxorubicin-induced alterations in kidney functioning, oxidative stress, DNA damage, and renal tissue morphology; Improvement by Acacia hydaspica tannin-rich ethyl acetate fraction. Saudi J. Biol. Sci. 27, 2251–2260. 10.1016/j.sjbs.2020.07.011 32884406 PMC7451730

[B2] Alharbi I. Alharbi H. Almogbel Y. Alalwan A. Alhowail A. (2020). Effect of metformin on doxorubicin-induced memory dysfunction. Brain Sci. 10, 152. 10.3390/brainsci10030152 32156040 PMC7139300

[B3] Alhowail A. (2021). Molecular insights into the benefits of nicotine on memory and cognition (Review). Mol. Med. Rep. 23, 398. 10.3892/mmr.2021.12037 33786606 PMC8025477

[B4] Alhowail A. (2022). Mechanisms underlying cognitive impairment induced by prenatal alcohol exposure. Brain Sci. 12, 1667. 10.3390/brainsci12121667 36552126 PMC9775935

[B5] Alhowail A. H. Almogbel Y. S. Abdellatif A. A. H. Aldubayan M. A. Chigurupati S. Nemala R. A. (2022). Effects of CMF and MET on glutamate and dopamine levels in the brain, and their impact on cognitive function. Eur. Rev. Med. Pharmacol. Sci. 26, 2353–2362. 10.26355/eurrev_202204_28465 35442489

[B6] Alhowail A. H. Bloemer J. Majrashi M. Pinky P. D. Bhattacharya S. Yongli Z. (2019a). Doxorubicin-induced neurotoxicity is associated with acute alterations in synaptic plasticity, apoptosis, and lipid peroxidation. Toxicol. Mech. methods 29, 457–466. 10.1080/15376516.2019.1600086 31010378

[B7] Alhowail A. H. Chigurupati S. Sajid S. Mani V. (2019b). Ameliorative effect of metformin on cyclophosphamide-induced memory impairment in mice. Eur. Rev. Med. Pharmacol. Sci. 23, 9660–9666. 10.26355/eurrev_201911_19460 31773717

[B8] Alhowail A. H. Pinky P. D. Eggert M. Bloemer J. Woodie L. N. Buabeid M. A. (2021). Doxorubicin induces dysregulation of AMPA receptor and impairs hippocampal synaptic plasticity leading to learning and memory deficits. Heliyon 7, e07456. 10.1016/j.heliyon.2021.e07456 34296005 PMC8282984

[B9] Alsaud M. M. Alhowail A. H. Aldubayan M. A. Almami I. S. (2023). The ameliorative effect of pioglitazone against neuroinflammation caused by doxorubicin in rats. Molecules 28, 4775. 10.3390/molecules28124775 37375330 PMC10304172

[B10] An J. Chen Y. Huang Z. (2004). Critical upstream signals of cytochrome c release induced by a novel bcl-2 inhibitor. J. Biol. Chem. 279, 19133–19140. 10.1074/jbc.M400295200 14966123

[B11] Bhattacharya S. Kimble W. Buabeid M. Bhattacharya D. Bloemer J. Alhowail A. (2017). Altered AMPA receptor expression plays an important role in inducing bidirectional synaptic plasticity during contextual fear memory reconsolidation. Neurobiol. Learn. Mem. 139, 98–108. 10.1016/j.nlm.2016.12.013 28034784

[B12] Bredlau A. L. Motamarry A. Chen C. McCrackin M. A. Helke K. Armeson K. E. (2018). Localized delivery of therapeutic doxorubicin dose across the canine blood-brain barrier with hyperthermia and temperature sensitive liposomes. Drug Deliv. 25, 973–984. 10.1080/10717544.2018.1461280 29688083 PMC6058514

[B13] Brothwell S. L. C. Barber J. L. Monaghan D. T. Jane D. E. Gibb A. J. Jones S. (2008). NR2B- and NR2D-containing synaptic NMDA receptors in developing rat substantia nigra pars compacta dopaminergic neurones. J. Physiology 586, 739–750. 10.1113/jphysiol.2007.144618 PMC2375608 18033813

[B14] Coelho-Santos V. Leitão R. A. Cardoso F. L. Palmela I. Rito M. Barbosa M. (2015). The TNF-α/NF-κB signaling pathway has a key role in methamphetamine-induced blood-brain barrier dysfunction. J. Cereb. Blood Flow Metabolism 35, 1260–1271. 10.1038/jcbfm.2015.59 PMC4528012 25899299

[B15] Crupi R. Impellizzeri D. Cuzzocrea S. (2019). Role of metabotropic glutamate receptors in neurological disorders. Front. Mol. Neurosci. 12, 20. 10.3389/fnmol.2019.00020 30800054 PMC6375857

[B16] Dong X.-x. Wang Y. Qin Z.-h. (2009). Molecular mechanisms of excitotoxicity and their relevance to pathogenesis of neurodegenerative diseases. Acta Pharmacol. Sin. 30, 379–387. 10.1038/aps.2009.24 19343058 PMC4002277

[B17] Gao C. Wang A.-Y. (2009). Significance of increased apoptosis and Bax expression in human small intestinal adenocarcinoma. J. Histochem. Cytochem. 57, 1139–1148. 10.1369/jhc.2009.954446 19729672 PMC2778087

[B18] Hardwick J. M. Soane L. (2013). Multiple functions of BCL-2 family proteins. Cold Spring Harb. Perspect. Biol. 5, a008722. 10.1101/cshperspect.a008722 23378584 PMC3552500

[B19] Hsieh Y.-H. Wang Q. Zhang L. Yuan X. Ou Y. Zhu X. (2016). The relationship between the bcl-2/bax proteins and the mitochondria-mediated apoptosis pathway in the differentiation of adipose-derived stromal cells into neurons. Plos One 11, e0163327. 10.1371/journal.pone.0163327 27706181 PMC5051896

[B20] Huang X. Hussain B. Chang J. (2020). Peripheral inflammation and blood–brain barrier disruption: effects and mechanisms. CNS Neurosci. Ther. 27, 36–47. 10.1111/cns.13569 33381913 PMC7804893

[B21] Kampa B. M. Clements J. Jonas P. Stuart G. J. (2004). Kinetics of Mg2+ unblock of NMDA receptors: implications for spike-timing dependent synaptic plasticity. J. Physiol. 556, 337–345. 10.1113/jphysiol.2003.058842 14754998 PMC1664940

[B22] Keeney J. T. R. Ren X. Warrier G. Noel T. Powell D. K. Brelsfoard J. M. (2018). Doxorubicin-induced elevated oxidative stress and neurochemical alterations in brain and cognitive decline: protection by MESNA and insights into mechanisms of chemotherapy-induced cognitive impairment (“chemobrain”). Oncotarget 9, 30324–30339. 10.18632/oncotarget.25718 30100992 PMC6084398

[B23] Kitamura Y. Hattori S. Yoneda S. Watanabe S. Kanemoto E. Sugimoto M. (2015). Doxorubicin and cyclophosphamide treatment produces anxiety-like behavior and spatial cognition impairment in rats: possible involvement of hippocampal neurogenesis via brain-derived neurotrophic factor and cyclin D1 regulation. Behav. Brain Res. 292, 184–193. 10.1016/j.bbr.2015.06.007 26057360

[B24] Li F. Tsien J. Z. (2009). Memory and the NMDA receptors. N. Engl. J. Med. 361, 302–303. 10.1056/NEJMcibr0902052 19605837 PMC3703758

[B25] Li M. H. Jang J. H. Surh Y. J. (2005). Nitric oxide induces apoptosis via AP-1-driven upregulation of COX-2 in rat pheochromocytoma cells. Free Radic. Biol. Med. 39, 890–899. 10.1016/j.freeradbiomed.2005.05.015 16140209

[B26] Lieu E. L. Nguyen T. Rhyne S. Kim J. (2020). Amino acids in cancer. Exp. Mol. Med. 52, 15–30. 10.1038/s12276-020-0375-3 31980738 PMC7000687

[B27] Livingstone R. W. Elder M. K. Singh A. Westlake C. M. Tate W. P. Abraham W. C. (2021). Secreted amyloid precursor protein-alpha enhances LTP through the synthesis and trafficking of Ca2+-permeable AMPA receptors. Front. Mol. Neurosci. 14, 660208. 10.3389/fnmol.2021.660208 33867938 PMC8047154

[B28] Long H.-Z. Cheng Y. Zhou Z.-W. Luo H.-Y. Wen D.-D. Gao L.-C. (2021). PI3K/AKT signal pathway: a target of natural products in the prevention and treatment of Alzheimer’s disease and Parkinson’s disease. Front. Pharmacol. 12, 648636. 10.3389/fphar.2021.648636 33935751 PMC8082498

[B29] Luscher C. Malenka R. C. (2012). NMDA receptor-dependent long-term potentiation and long-term depression (LTP/LTD). Cold Spring Harb. Perspect. Biol. 4, a005710. 10.1101/cshperspect.a005710 22510460 PMC3367554

[B30] Mizuta T. Shimizu S. Matsuoka Y. Nakagawa T. Tsujimoto Y. (2007). A bax/bak-independent mechanism of cytochrome c release. J. Biol. Chem. 282, 16623–16630. 10.1074/jbc.M611060200 17409097

[B31] Naseri M. H. Mahdavi M. Davoodi J. Tackallou S. H. Goudarzvand M. Neishabouri S. H. (2015). Up regulation of Bax and down regulation of Bcl2 during 3-NC mediated apoptosis in human cancer cells. Cancer Cell Int. 15, 55. 10.1186/s12935-015-0204-2 26074734 PMC4464715

[B32] Ndountse L. T. Chan H. M. (2009). Role of N-methyl-D-aspartate receptors in polychlorinated biphenyl mediated neurotoxicity. Toxicol. Lett. 184, 50–55. 10.1016/j.toxlet.2008.10.013 19022367

[B33] Newcomer J. W. Farber N. B. Olney J. W. (2022). NMDA receptor function, memory, and brain aging. Dialogues Clin. Neurosci. 2, 219–232. 10.31887/DCNS.2000.2.3/jnewcomer PMC3181613 22034391

[B34] Park M. H. Hong J. T. (2016). Roles of NF-κB in cancer and inflammatory diseases and their therapeutic approaches. Cells 5, 15. 10.3390/cells5020015 27043634 PMC4931664

[B35] Pimentel C. Batista-Nascimento L. Rodrigues-Pousada C. Menezes R. A. (2012). Oxidative stress in Alzheimer's and Parkinson's diseases: insights from the YeastSaccharomyces cerevisiae. Oxidative Med. Cell. Longev. 2012, 132146–132149. 10.1155/2012/132146 PMC3371773 22701754

[B36] Poligone B. Baldwin A. S. (2001). Positive and negative regulation of NF-kappaB by COX-2: roles of different prostaglandins. J. Biol. Chem. 276, 38658–38664. 10.1074/jbc.M106599200 11509575

[B37] Polli F. S. Kohlmeier K. A. (2022). Prenatal nicotine alters development of the laterodorsal tegmentum: possible role for attention-deficit/hyperactivity disorder and drug dependence. World J. Psychiatry 12, 212–235. 10.5498/wjp.v12.i2.212 35317337 PMC8900586

[B38] Qu W. Yuan B. Liu J. Liu Q. Zhang X. Cui R. (2020). Emerging role of AMPA receptor subunit GluA1 in synaptic plasticity: implications for Alzheimer's disease. Cell Prolif. 54, e12959. 10.1111/cpr.12959 33188547 PMC7791177

[B39] Shamas-Din A. Kale J. Leber B. Andrews D. W. (2013). Mechanisms of action of bcl-2 family proteins. Cold Spring Harb. Perspect. Biol. 5, a008714. 10.1101/cshperspect.a008714 23545417 PMC3683897

[B40] Sharifi S. Barar J. Hejazi M. S. Samadi N. (2015). Doxorubicin changes Bax/Bcl-xL ratio, caspase-8 and 9 in breast cancer cells. Adv. Pharm. Bull. 5, 351–359. 10.15171/apb.2015.049 26504757 PMC4616902

[B41] Singh L. Pushker N. Saini N. Sen S. Sharma A. Bakhshi S. (2015). Expression of pro-apoptotic Bax and anti-apoptotic Bcl-2 proteins in human retinoblastoma. Clin. Exp. Ophthalmol. 43, 259–267. 10.1111/ceo.12397 25132102

[B42] Singh R. Letai A. Sarosiek K. (2019). Regulation of apoptosis in health and disease: the balancing act of BCL-2 family proteins. Nat. Rev. Mol. Cell Biol. 20, 175–193. 10.1038/s41580-018-0089-8 30655609 PMC7325303

[B43] Song S. Chu L. Liang H. Chen J. Liang J. Huang Z. (2019). Protective effects of dioscin against doxorubicin-induced hepatotoxicity via regulation of sirt1/FOXO1/NF-κb signal. Front. Pharmacol. 10, 1030. 10.3389/fphar.2019.01030 31572199 PMC6753638

[B44] Su L.-J. Zhang J.-H. Gomez H. Murugan R. Hong X. Xu D. (2019). Reactive oxygen species-induced lipid peroxidation in apoptosis, autophagy, and ferroptosis. Oxidative Med. Cell. Longev. 2019, 5080843–5080913. 10.1155/2019/5080843 PMC6815535 31737171

[B45] Suzuki Y. Nakamoto C. Watanabe-Iida I. Watanabe M. Takeuchi T. Sasaoka T. (2023). Quantitative analysis of NMDA receptor subunits proteins in mouse brain. Neurochem. Int. 165, 105517. 10.1016/j.neuint.2023.105517 36913980

[B46] Thomas T. C. Beitchman J. A. Pomerleau F. Noel T. Jungsuwadee P. Butterfield D. A. (2017). Acute treatment with doxorubicin affects glutamate neurotransmission in the mouse frontal cortex and hippocampus. Brain Res. 1672, 10–17. 10.1016/j.brainres.2017.07.003 28705715 PMC5576558

[B47] Thorn C. F. Oshiro C. Marsh S. Hernandez-Boussard T. McLeod H. Klein T. E. (2011). Doxorubicin pathways: pharmacodynamics and adverse effects. Pharmacogenetics Genomics 21, 440–446. 10.1097/FPC.0b013e32833ffb56 21048526 PMC3116111

[B48] Traynelis S. F. Wollmuth L. P. McBain C. J. Menniti F. S. Vance K. M. Ogden K. K. (2010). Glutamate receptor ion channels: structure, regulation, and function. Pharmacol. Rev. 62, 405–496. 10.1124/pr.109.002451 20716669 PMC2964903

[B49] Ureshino R. P. Erustes A. G. Bassani T. B. Wachilewski P. Guarache G. C. Nascimento A. C. (2019). The interplay between Ca2+ signaling pathways and neurodegeneration. Int. J. Mol. Sci. 20, 6004. 10.3390/ijms20236004 31795242 PMC6928941

[B50] Usmani M. T. Krattli R. P. El-Khatib S. M. Le A. C. D. Smith S. M. Baulch J. E. (2023). BDNF augmentation using riluzole reverses doxorubicin-induced decline in cognitive function and neurogenesis. Neurotherapeutics 20, 838–852. 10.1007/s13311-022-01339-z 36720792 PMC10275819

[B51] Wang C. Youle R. J. (2009). The role of mitochondria in apoptosis. Annu. Rev. Genet. 43, 95–118. 10.1146/annurev-genet-102108-134850 19659442 PMC4762029

[B52] Wolfrum P. Fietz A. Schnichels S. Hurst J. (2022). The function of p53 and its role in Alzheimer’s and Parkinson’s disease compared to age-related macular degeneration. Front. Neurosci. 16, 1029473. 10.3389/fnins.2022.1029473 36620455 PMC9811148

[B53] Wright A. Vissel B. (2012). The essential role of AMPA receptor GluR2 subunit RNA editing in the normal and diseased brain. Front. Mol. Neurosci. 5, 34. 10.3389/fnmol.2012.00034 22514516 PMC3324117

[B54] Yang X. Gong R. Qin L. Bao Y. Fu Y. Gao S. (2022). Trafficking of NMDA receptors is essential for hippocampal synaptic plasticity and memory consolidation. Cell Rep. 40, 111217. 10.1016/j.celrep.2022.111217 35977502

[B55] Yang Y. Ju W. Zhang H. Sun L. (2018). Effect of ketamine on LTP and NMDAR EPSC in Hippocampus of the chronic social defeat stress mice model of depression. Front. Behav. Neurosci. 12, 229. 10.3389/fnbeh.2018.00229 30356718 PMC6189398

[B56] Zanetti L. Regoni M. Ratti E. Valtorta F. Sassone J. (2021). Presynaptic AMPA receptors in health and disease. Cells 10, 2260. 10.3390/cells10092260 34571906 PMC8470629

[B57] Zhao L. Zhang B. (2017). Doxorubicin induces cardiotoxicity through upregulation of death receptors mediated apoptosis in cardiomyocytes. Sci. Rep. 7, 44735. 10.1038/srep44735 28300219 PMC5353581

[B58] Zhou Y. Danbolt N. C. (2014). Glutamate as a neurotransmitter in the healthy brain. J. Neural Transm. 121, 799–817. 10.1007/s00702-014-1180-8 24578174 PMC4133642

